# DJ-1 promotes epithelial-to-mesenchymal transition via enhancing FGF9 expression in colorectal cancer

**DOI:** 10.1242/bio.051680

**Published:** 2020-05-21

**Authors:** Longhao Li, Chundong Zhang, Yi Li, Ying Zhang, Yunlong Lei

**Affiliations:** 1 Department of Oncology, The First Affiliated Hospital of Chongqing Medical University, Chongqing 400016, China; 2 Department of Biochemistry and Molecular Biology, and Molecular Medicine and Cancer Research Center, Chongqing Medical University, Chongqing 400016, China

**Keywords:** Colorectal cancer, DJ-1, FGF9, EMT

## Abstract

Tumor metastasis is the main contributor to high recurrence and mortality in colorectal cancer (CRC). In a previous study, we found that DJ-1 plays an important role in CRC metastasis, and is the main target in Ciclopirox olamine (CPX)-treated CRC. However, the mechanism underlying DJ-1-induced CRC metastasis remains elusive. In the present study, our results showed that DJ-1 could activate Wnt signaling resulting in enhanced invasive potential and epithelial-to-mesenchymal transition (EMT) in CRC cells. RNA-seq and bioinformatics analysis reveals that the DJ-1/Wnt signaling pathway may promote CRC cells’ EMT by regulating fibroblast growth factor 9 (FGF9) expression. Molecular validation showed that expression of FGF9 was upregulated by the DJ-1/Wnt signaling pathway and decreasing FGF9-expression impeded DJ-1-induced CRC invasive ability and EMT, suggesting that FGF9 is involved in DJ-1-enhanced CRC metastasis. In addition, we show that FGF9 was overexpressed in CRC human specimens and was significantly associated with tumor differentiation. High FGF9 expression was correlated with worse overall survival, and a correlation exhibited between FGF9 and EMT markers (E-cadherin and Vimentin) in CRC samples. Together, our results determined that FGF9 was involved in DJ-1-induced invasion and EMT in CRC cells, and may represent a promising therapeutic candidate for CRC anti-metastatic strategies.

## Abstract (precis)

**Summary:** DJ-1 could activate Wnt signaling to enhance FGF9 expression, resulting in enhanced invasive potential and epithelial-to-mesenchymal transition (EMT) in CRC cells. FGF9 predicts poor survival in patients with CRC.

## INTRODUCTION

Colorectal cancer (CRC) represents the third most common cancer and the second highest cancer-related cause of death worldwide (Bray et al., 2018). Metastasis is mainly involved in treatment failure and CRC-related death: the 5-year survival rate of CRC patients who are diagnosed at an early stage is 90%, while it is only 14% for people in whom the cancer extends beyond the colon (Siegel et al., 2017). Therefore, revealing the detailed molecular mechanisms of metastasis is important for the treatment of patients with metastatic CRC.

DJ-1, involved in multiple biological processes of cells and largely associated with Parkinson's disease, is initially cloned as a putative oncogene (Cao et al., 2015). Over-expression of DJ-1 is found in various cancers and is closely associated with tumor progression (Cao et al., 2015; He et al., 2012). We previously found that DJ-1 could promote CRC progression and is significantly associated with poor patient survival (Zhou et al., 2018). In addition, we found that a synthetic antifungal agent (Ciclopirox olamine, CPX), which has been used for treating advanced solid tumors in a United States multi-center first-in-human Phase 1 clinical trial (NCT03348514), exhibits considerable anti-CRC efficacy by targeting DJ-1 (Zhou et al., 2019), suggesting the potential of targeting DJ-1 for treating CRC. Thus, it is important to reveal the mechanisms of DJ-1 contributing to CRC progression.

Epithelial-to-mesenchymal transition (EMT) is a critical process during tumor metastasis, in which stationary epithelial cells are converted to invasive mesenchymal cells by inhibiting E-cadherin-based cell adhesion and enhancing mesenchymal marker expression, including Vimentin, N-cadherin and Fibronectin (Brabletz et al., 2018; Vu and Datta, 2017). The fibroblast growth factors and their receptors (FGF/FGFR) have been widely reported to be involved in proliferation and differentiation of primary epithelial cells; thus, deregulation of FGF signaling is largely linked to tumor initiation and progression (Ghedini et al., 2018; Zhang et al., 2019), especially in EMT program (Liu et al., 2015, 2013).

In the present study, our results demonstrate that DJ-1 could induce EMT of CRC cells through activating Wnt/FGF9 signaling. In addition, advanced CRCs have higher expression of FGF9, which is associated with poor outcomes in CRC patients. Collectively, our results show that FGF9 plays an important role in the DJ-1/Wnt signaling pathway-induced CRC metastasis process.

## RESULTS

### DJ-1 induces EMT in CRC

**Fig. 1.**
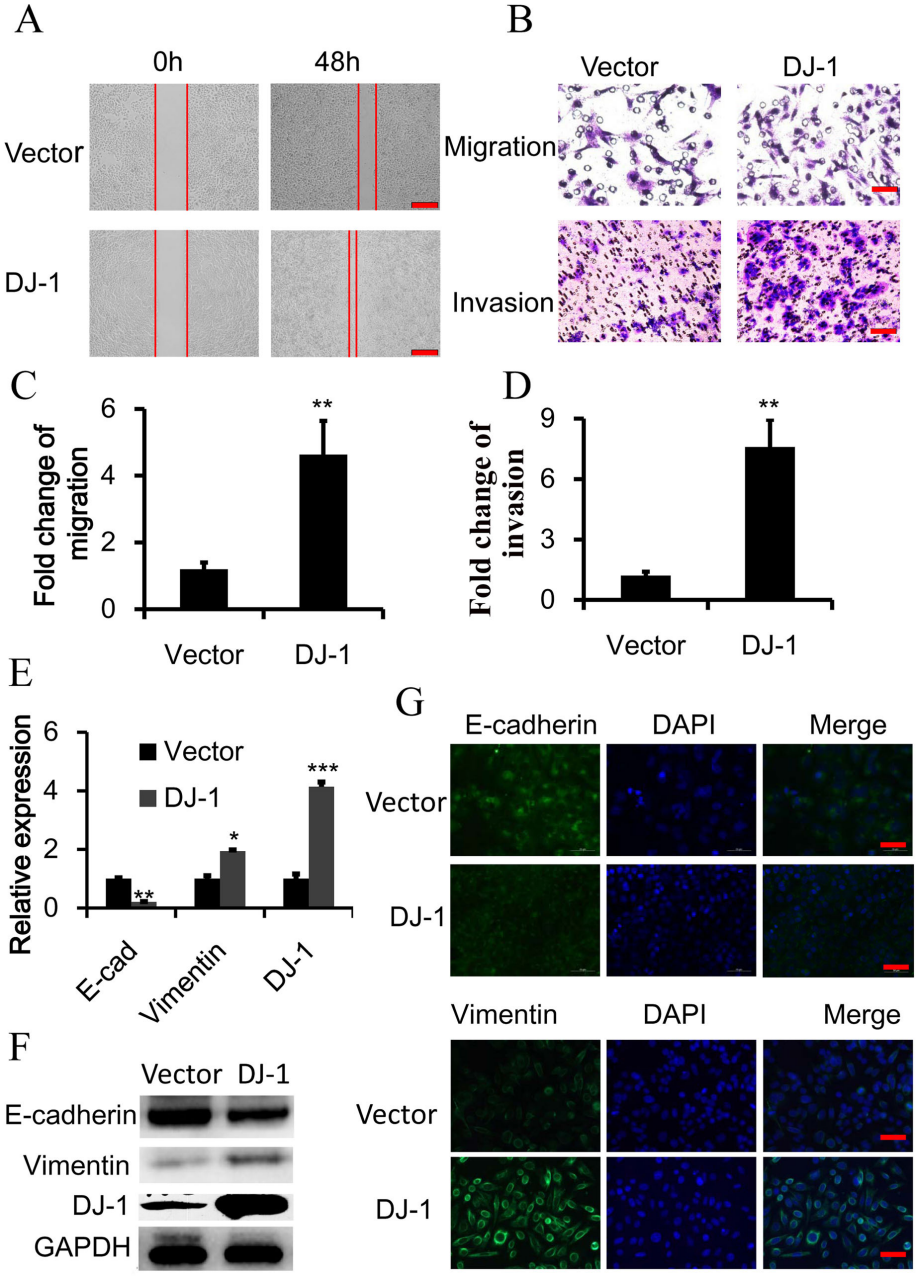
**DJ-1 induces EMT in colorectal cancer cells.** DJ-1 was stably overexpressed in SW480 cells. (A) Wound-healing migration assay of indicated time. Scale bars: 250 μm. (B–D) Quantitative analysis of cell migration and Matrigel invasion assays. Migration was analyzed at 24 h, invasion at 48 h. All data were from at least three independent experiments and shown as mean±s.d. Scale bars: 50 μm. (E) Expression of DJ-1, E-cadherin and Vimentin was examined by qRT-PCR. Y-axis means relative expression to control group. (F) Expression of DJ-1, E-cadherin and Vimentin was examined by immunoblot. (G) Expression of E-cadherin and Vimentin was examined by immunofluorescence. Scale bars: 50 μm. *P<0.05, **P<0.01, ***P<0.001.

To further reveal the mechanisms of DJ-induced CRC metastasis, SW480 cells were stably transfected with DJ-1 cDNA and then their migratory and invasive capacity was evaluated. As shown as Fig. 1A, wound closure in DJ-1 cDNA-transfected SW480 cells was faster than in SW480 cells with vector in wound-healing migration assays. Similarly, exogenous DJ-1 expression obviously promoted the migration and invasion of SW480 cells in Transwell migration and Matrigel invasion assessments (Fig. 1B–D). Considering EMT is an important step in the process of cancer invasion and metastasis, we examined whether DJ-1 could alter the expression of the EMT markers E-cadherin and Vimentin. Interestingly, DJ-1 overexpression can promote EMT in SW480 cells, evidenced by a reduction of E-cadherin (the classic epithelial marker) and upregulation of Vimentin (a classic mesenchymal marker) in transcription and protein level (Fig. 1E,F). Consistent with this result, immunofluorescence analysis also showed that DJ-1 could repress the expression of E-cadherin on the cell surface while promoting Vimentin expression in whole cells (Fig. 1G). Collectively, these results show that DJ-1 can promote CRC cell invasion by inducing EMT.

### DJ-1 induces EMT by activating Wnt signaling

**Fig. 2.**
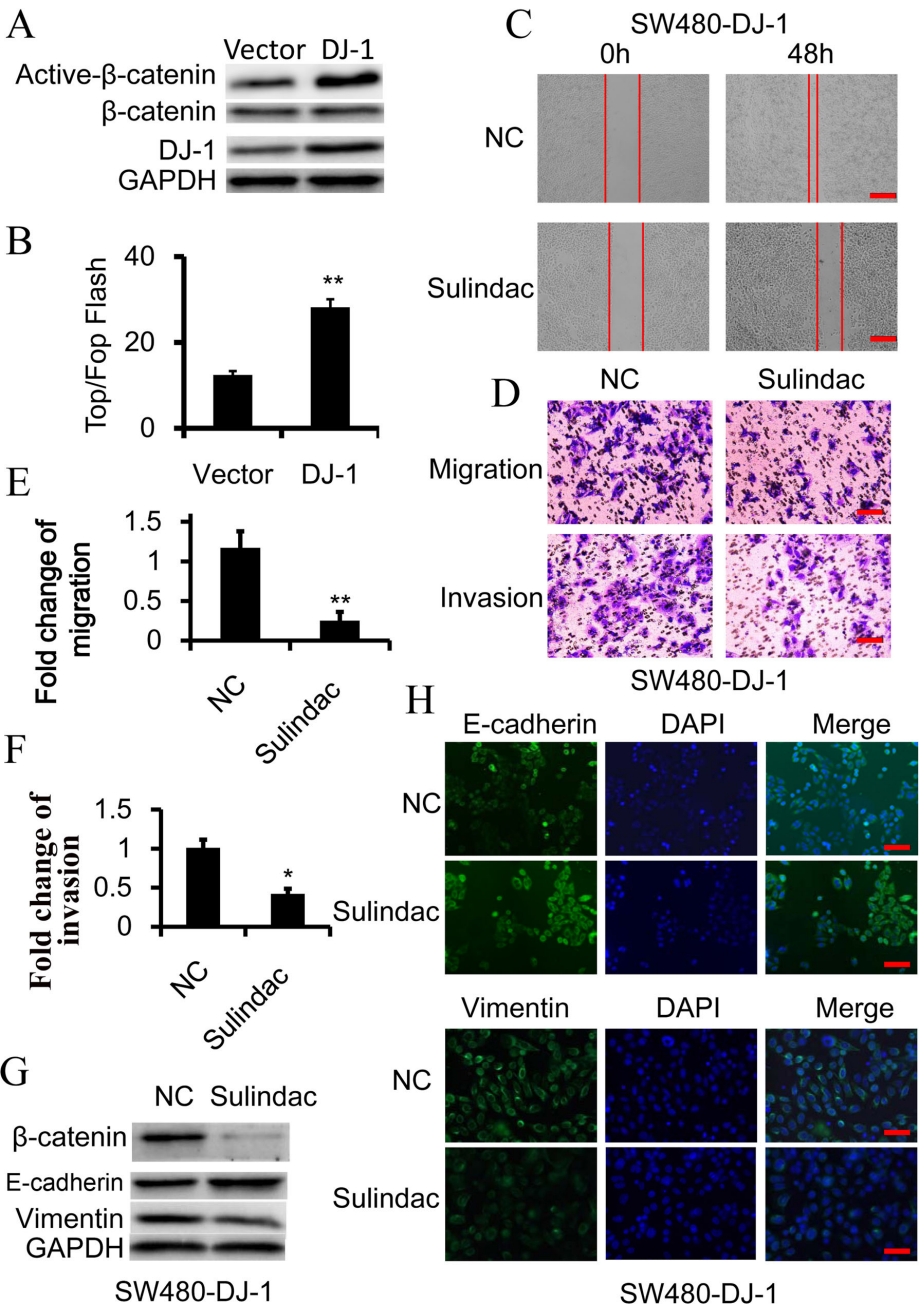
**Wnt signaling is essential for DJ-1-induced EMT.** (A) Western blot analysis of β-catenin and activated-β-catenin (non-phosphorylated β-catenin) expression in SW480 cells, in which DJ-1 was overexpressed. (B) TOP-Flash/FOP-Flash assay depicting Wnt signaling activity in SW480 and DJ-1-overexpressed SW480 (SW480-DJ-1) cells. (C–H) SW480-DJ-1 cells were treated with 100 μM Wnt inhibitor Sulindac for 36 h. (C) Wound-healing migration assay of indicated time. Scale bars: 250 μm. (D–F) Quantitative analysis of cell migration and Matrigel invasion assays. Migration was analyzed at 24 h, invasion at 48 h. All data were from at least three independent experiments and shown as mean±s.d. Scale bars: 50 μm. (G) Expression of β-catenin, E-cadherin, and Vimentin was examined by immunoblot. (H) Expression of E-cadherin and vimentin was examined by immunofluorescence. Scale bars: 50 μm. *P<0.05, **P<0.01.

The Wnt-signaling pathway is most associated with CRC initiation and progression, and a number of studies have demonstrated that abnormal activation of the Wnt-signaling pathway could induce metastasis and EMT in CRC (Zhou et al., 2018; Chen et al., 2016; White et al., 2012; Wu et al., 2013). Thus, we assess the potential role of Wnt signaling in DJ-1-mediated invasion and EMT of CRC. As expected, DJ-1 overexpression could promote the expression of activated (non-phosphorylated) β-catenin and enhance the activity of TCF/LEF transcription in SW480 cells, suggesting that Wnt signaling could be activated by DJ-1 (Fig. 2A,B). To determine whether Wnt signaling was required in DJ-1-mediated invasion and EMT of CRC cells, sulindac, a Wnt signaling inhibitor, was employed to treat SW480-DJ-1 cells by degrading β-catenin. The results showed that inhibition of Wnt signaling effectively restrained the increased invasive and migratory ability induced by DJ-1 (Fig. 2C­–F). Correspondingly, sulindac treatment could restore E-cadherin expression and inhibit Vimentin overexpression in DJ-1 overexpressed SW480 cells (Fig. 2G,H; Fig. S1). These results indicated that DJ-1 induces EMT by activating Wnt signaling in CRC cells.

### FGF9 is vital for DJ-1/Wnt-induced EMT

**Fig. 3.**
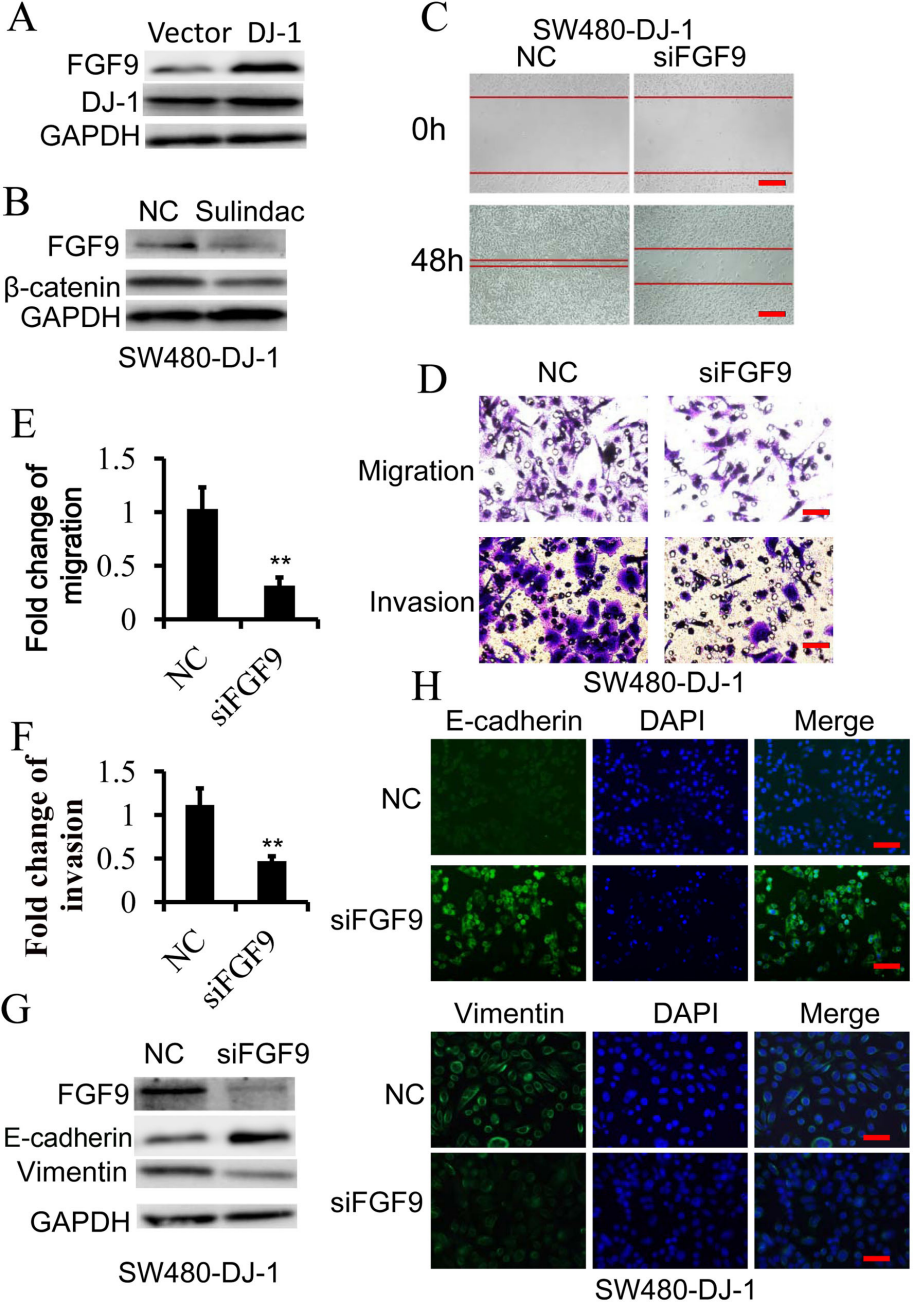
**FGF9 is required for DJ-1-induced and Wnt signaling-mediated EMT.** (A) Western blot analysis of FGF9 expression in SW480 cells stable transfected with DJ-1 cDNA or vector. (B) Western blot analysis of FGF9 in SW480-DJ-1 cells treated with or without 100 μM Sulindac for 36 h. (C–H) SW480-DJ-1 cells were transfected with specific FGF9 siRNA. (C) Wound-healing migration assay of indicated time. Scale bars: 250 μm. (D–F) Quantitative analysis of cell migration and Matrigel invasion assays. Migration was analyzed at 24 h, invasion at 48 h. All data were from at least three independent experiments and shown as mean±s.d. Scale bars: 50 μm. (G) Expression of FGF9, E-cadherin, and Vimentin was examined by immunoblot. (H) Expression of E-cadherin and Vimentin was examined by Immunofluorescence. Scale bars: 50 μm. **P<0.01.

In our previous study, the results of our RNA sequences and bioinformatics analysis showed that the expression of 27 targeted genes in Wnt signaling was enhanced more than onefold after DJ-1 overexpression in CRC cells (Zhou et al., 2018). Of those genes, the expression level of FGF9 was one of the most significantly upregulated after DJ-1 overexpression. In fact, DJ-1 can indeed promote transcription and expression of FGF9 in SW480 cells (Fig. 3A; Fig. S2A), while sulindac-induced Wnt signaling inactivation could suppress FGF9 transcription and expression in DJ-1-overexpressed SW480 cells (Fig. 3B; Fig. S2B), suggesting that DJ-1 could enhance FGF9 expression by activating Wnt signaling. To determine the role of FGF9 in DJ-1/Wnt signaling-mediated EMT of CRC, FGF9 was knocked down by siRNA in SW480 cells with DJ-1 overexpression, and then wound-healing migration assays, Transwell migration and Matrigel invasion assays were performed. The results showed that DJ-1-enhanced migratory and invasive capacity was markedly retarded after knockdown of FGF9 in CRC cells (Fig. 3C–F). In line with this, repression of FGF9 expression abrogated the DJ-1-initiated EMT process of SW480 cells (Fig. 3G,H; Fig. S2C). To rule out the potential cell type-specific effect, we further examined the role of DJ-1-inducing EMT by upregulating FGF9 in another CRC cell line, RKO. As expected, DJ-1 also could promote FGF9 expression, induce EMT and significantly enhance the migratory and invasive ability in RKO cells, while FGF9 knockdown would reverse these effects (Fig. S3). These results demonstrate that FGF9 is vital for DJ-1/Wnt-induced EMT in CRC cells.

### FGF9 predicts poor survival in patients with CRC

**Fig. 4.**
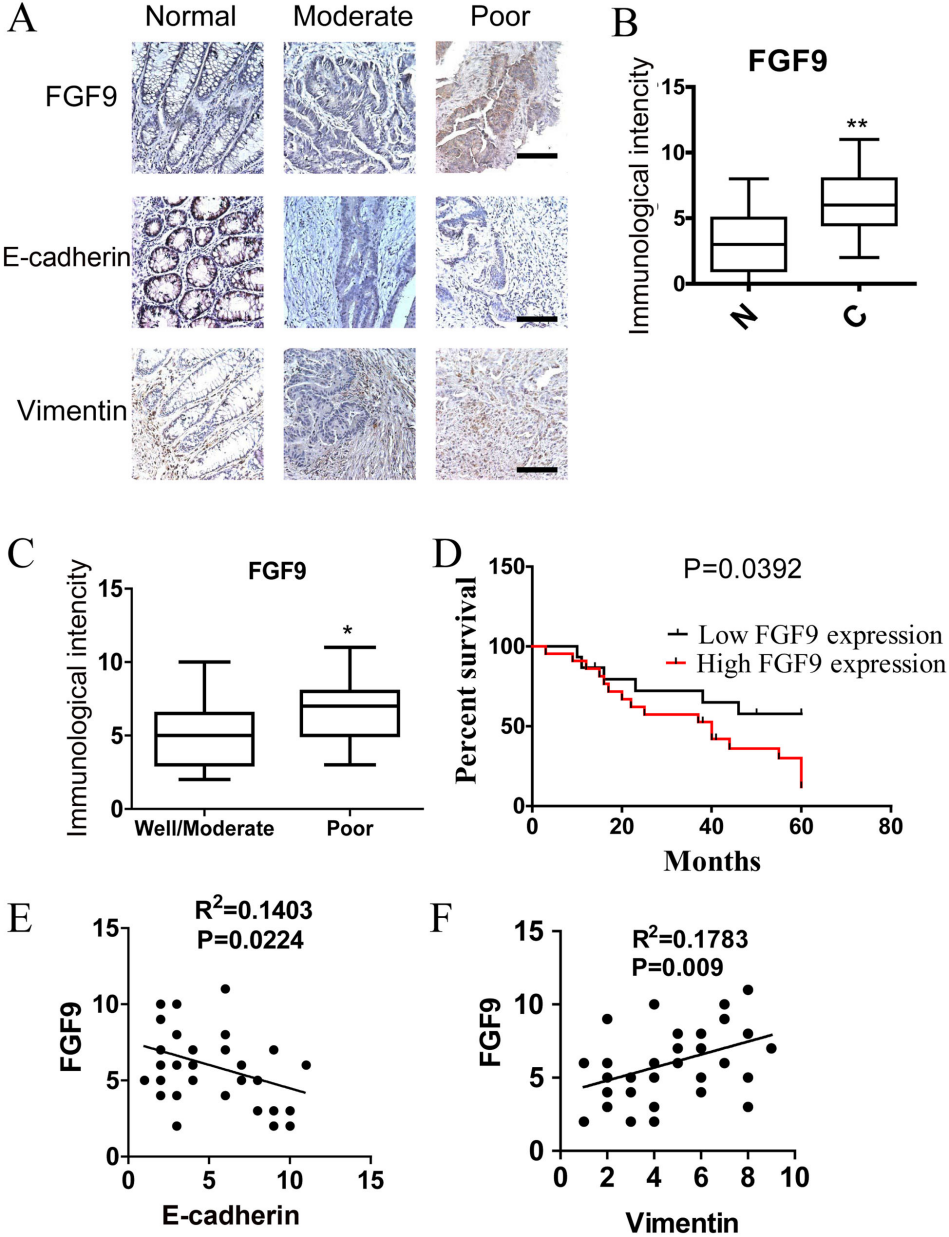
**FGF9 predicts poor survival in patients with CRC.** (A) Immunohistochemical staining of FGF9, E-cadherin and Vimentin in paraffin-embedded human CRC tissues. Scale bars: 25 μm. (B) Immunohistochemical scores for FGF9 in normal colorectal mucosa and CRC tissues (*n*=37). (C) Immunohistochemical staining of FGF9 in well-, moderately and poorly differentiated CRC tissues. (D) Kaplan–Meier survival curves of CRC patients with low (*n*=15) and high (*n*=22) FGF9 expression. FGF9 was classified as high expression if the immunostaining score is more than four, otherwise classified as low expression. (E) Correlation test of immunostaining intensity between FGF9 and E-cadherin. (F) Correlation test of immunostaining intensity between FGF9 and Vimentin. *P<0.05, **P<0.01.

We next examined the expression partners of DJ-1 and FGF9 in 37 CRC specimens and their paired adjacent normal tissues by immunohistochemical staining. Corresponding with our previous study (Zhou et al., 2018), the expression of DJ-1 is significantly higher in CRC specimens compared with paired normal tissues (Fig. S4A,B, *P*<0.01). While the intensity of staining for FGF9 in CRC specimens was also significantly higher than that in matched normal tissues (Fig. 4A,B, *P*<0.001) and is closely associated with DJ-1 expression (R^2^=0.1529, *P*=0.0167) (Fig. S4C), demonstrating that FGF9 is overexpressed in human CRC. By analyzing the relationship between the expression levels of FGF9 in CRC with various clinical-pathological parameters, we found that FGF9 expression was inversely associated with histodifferentiation (*P*<0.05, Fig. 4C). Furthermore, the 37 colorectal carcinoma specimens were stratified as 22 high- and 15 low-FGF9-expressing tumors (FGF9 was classified as high expression if the immunostaining score was more than four, otherwise it was classified as low expression). A Kaplan–Meier survival analysis showed that CRC patients with high FGF9 expression have a poor outcome (Fig. 4D). Interestingly, as indicated as Fig. 4A, our results also suggested that higher Vimentin and lower E-cadherin levels were usually observed in CRC specimens with high FGF9 expression. Indeed, the protein expression of FGF9 is closely associated with Vimentin (R^2^=0.1783, *P*=0.009) and shows an inverse correlation with E-cadherin (R^2^=0.1403, *P*=0.0224) (Fig. 4E,F). However, no significant correlation was shown between FGF9 and DJ-1 with E-cadherin or Vimentin (Fig. S4D,E). These results suggest that FGF9 plays an important role in the progression and EMT of CRC.

## DISCUSSION

Despite the great progress in CRC diagnosis and treatment in recent years, including novel chemotherapies and targeted drugs, metastasis and postoperative recurrence still are the main cause of CRC deaths (Siegel et al., 2017). We previously found that DJ-1 was a biomarker of poor outcomes in CRC patients and could induce an aggressive phenotype in CRC cells both *in vitro* and *in vivo* (Zhou et al., 2018). In addition, we also found that CPX could serve as an antitumor agent by targeting DJ-1 and inhibiting autophagy, which could significantly strengthen its anti-CRC effect (Zhou et al., 2019). However, the mechanism of how DJ-1 promotes CRC metastasis has not fully been revealed. In the present study, we found that DJ-1 could promote CRC cells’ EMT, which is a critical step in CRC progression and drug sensitivity. The evidence of DJ-1-inucing EMT is rare. Only two reports have shown that DJ-1 could promote EMT in breast cancer cells and human proximal tubular epithelial cells via repressing KLF17 expression or PTEN expression (Ismail et al., 2014; Yao et al., 2011).

The mutation, deregulated expression and post-modification of DJ-1 have been linked to various pathogenesis such as neurodegenerative diseases, tumors, male infertility and type 2 diabetes (Cao et al., 2015). DJ-1 was widely involved in multiple biological processes of cells including regulating oxidative stress, transcription, mitochondrial regulation, fertilization, apoptosis and autophagy (Chan and Chan, 2015; Vasseur et al., 2012). High DJ-1 expression has been found in many types of tumors, including uveal melanoma, pancreatic ductal adenocarcinoma, non-small cell lung carcinoma, esophageal squamous cell carcinoma, breast cancer, hepatocellular carcinoma and ovarian carcinoma, and is significantly correlated with metastasis or poor survival outcomes (Davidson et al., 2008; He et al., 2012; Kim et al., 2005; Sitaram et al., 2009). DJ-1 can promote tumor initiation and progression through multiple pathways such as activating HIFα, MEK/ERK, Akt/mTOR, NF-κB signaling pathway, or inhibiting JNK, p53 and ASK1 (Davidson et al., 2008; He et al., 2012; Kim et al., 2005; Sitaram et al., 2009). In this study, we found that DJ-1 could promote EMT by activating the Wnt signaling pathway, which has been widely accepted to be the main cause in the process of CRC metastasis including EMT.

FGF9 can be expressed by stromal cells or epithelial cells and plays an important role in development by promoting EMT in normal cells (Corn et al., 2013; Ghedini et al., 2018). FGF9 takes important mitogenic effects on a variety of cell types and is critical for lung development and bone repair (Corn et al., 2013; Cui et al., 2019). FGF9 is ubiquitously expressed in embryos, however, only a few adult organs can produce FGF9, including the uterus, kidneys and brain (Corn et al., 2013; Cui et al., 2019). FGF9 is first identified from the secretions of human glioma MCF-G1 cells, and could promote NIH-3T3 cell line malignant transformation, suggesting that FGF9 may be a tumor-promoting factor (Naruo et al., 1993). In fact, high FGF9 expression is found in various human tumors such as prostate cancer, non small-cell lung cancer, hepatocellular carcinoma and ovarian endometrioid adenocarcinom, and is significantly associated with the progression of these cancer cells (Cui et al., 2019; He et al., 2018; Wang et al., 2019). Furthermore, Wang et al. found that miRNA-214 contributes to EMT and metastasis by targeting FGF9 in gastric cancer cells (Wang et al., 2019). However, FGF9 has also been shown to suppress renal tumor and lung squamous cell carcinoma metastases (Wang et al., 2017; Yin et al., 2015). In this study, we found that FGF9 is a main target in DJ-1/Wnt signaling-induced CRC EMT, and FGF9 overexpression is closely associated with poor patient outcome. In line with our results, Chen et al. found that FGF9 was overexpressed in CRC, and hypoxia could promote its expression by translational activation (Chen et al., 2014). In addition, Mizukami et al. found that high FGF9 expression in CRC patients is associated with anti-EGFR therapies resistance and FGF9 amplification is frequently observed in more aggressive CRC tumors (Mizukami et al., 2017). More interestingly, the expression or mutation of FGF9 correlates with β-catenin in ovarian, colorectal and endometrial carcinomas (Abdel-Rahman et al., 2008; Hendrix et al., 2006).

### Conclusions

Collectively, our results have shown that DJ-1 activates Wnt signaling to enhance the expression of FGF9, leading to EMT of CRC cells. In addition, we have shown that the expression of FGF9 is closely associated with EMT markers in human CRC specimens and that CRC patients with high levels of FGF9 have a poor prognosis. The results shed light on the role and mechanisms of DJ-1-induced CRC metastasis.

## MATERIALS AND METHODS

### Clinical specimens and immunohistochemistry

37 human CRC tissues and corresponding normal tissues were collected from Sichuan Provincial People's Hospital of China. The information of clinicopathologic characteristics was obtained from Department of Pathology in Sichuan Provincial People and summarized in Table 1, which was determined by two experienced pathologists. The Institutional Ethics Committee of Chongqing medical University granted ethics approval. Patients or their relatives signed the informed consent for tissue procurement.

**Table 1.**
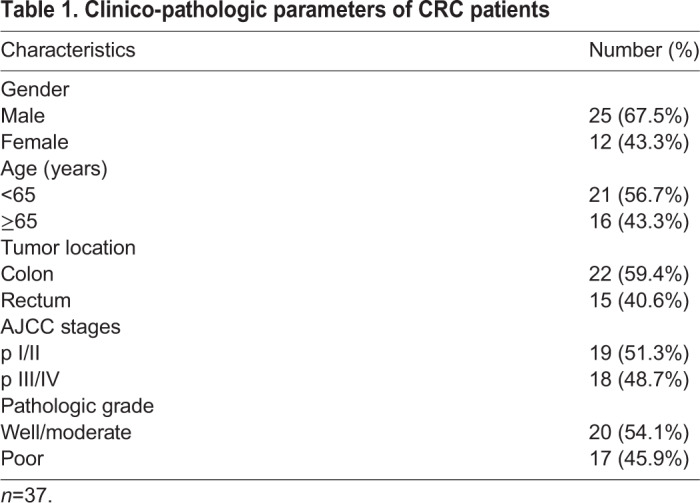
**Clinico-pathologic parameters of CRC patients**

Immunohistochemistry (IHC) and the immunostaining score for each slide were performed as described in our previous study (Liu et al., 2015). Genes were classified as high expression if their immunostaining score was more than four; otherwise they were classified as low expression.

### Cells and cell culture

The SW480 and RKO cell line was obtained from the American Type Culture Collection. In a humidified chamber, we cultured SW480 and RKO cells with Dulbecco's Modified Eagle's Medium supplement with streptomycin (10 mg/l), penicillin (10^7^ U/L) and fetal bovine serum (10%) at 5% CO_2_ and 37°C. As described in a previous study, the last time of authentication for these cells was May 2019 (Zhou et al., 2019).

As described in Boon et al., (2004), sulindac sulphide was prepared as 1000× stock solution in dimethyl sulphoxide (DMSO). Control cultures contained DMSO at an equivalent dilution, resulting in a final DMSO concentration of 0.1%. Cells were grown with or without the addition of sulindac sulphide (100 μM) for 36 h.

### Antibodies

Antibodies used in this study, including DJ-1 (SC-55573, 1:1500 for immunoblot, 1:200 for IHC) and Vimentin (SC-6260, 1:1000 for immunoblot, 1:100 for IHC and immunofluorescence), were obtained from Santa Cruz Biotechnology. GAPDH (D16H11) (#5174, 1:1500 for immunoblot), β-catenin (D10A8) (#8480, 1:800 for immunoblot) and non-phosphorylated (active) β-catenin (D13A1) (Ser33/37/Thr41; #8814, 1:1000 for immunoblot) were from Cell Signaling Technology. E-cadherin (ab15148, 1:1000 for immunoblot, 1:100 for IHC and immunofluorescence) was from Abcam.

### Transfection

FGF9 siRNA was obtained from GenePharma Company (Shanghai, China) and transfected with Lipofectamine RNAiMAX reagent (Invitrogen, USA). The sequence of the FGF9 siRNA is sense (5′-3′) GGACUAAACGGCACCAGAATT. DJ-1 cDNA was a gift from Professor Philipp J. Kahle at the University of Tübingen, Germany, and was transfected into the indicated cells using Lipofectamine 2000 Reagent (Invitrogen), following be selected under zeocin for 3 to 5 weeks for screening stably transfected clones.

### Quantitative RT-PCR (qRT-PCR)

Total RNA was extracted from cells using Trizol reagent (Invitrogen). cDNA reverse transcription, and qRT-PCR were performed as per the manufacturers’ instructions using Reverse Transcription PrimeScript 1st Stand cDNA Synthesis kit (TaKaRa, Otsu, Japan) and quantitative PCR reagents SYBR PremixEx TaqTM (TaKaRa). The relative expression of genes was calculated with the 2^−ΔΔCt^ method. The sequences of the primers used are presented in Table S1.

### Wound healing and transwell assays

In wound healing assay, a sterile pipet tip was used to create wounds in confluent cells and then to remove free-floating cells and debris. After 48 h, wound healing was documented and visualized. 24-well transwell chambers (Corning) were used to perform cell-migration assays. 3×10^4^ cells were seeded in the upper chamber and cultured with serum-free medium for 24 h, and then the number of cells in the lower chamber was counted. BD Matrigel (1:5) was added and incubated for 4 h in the transwell membrane chambers for cell-invasion assays. 8×10^4^ cells were seeded in the upper chamber and cultured with serum-free medium for 48 h and then the number of cells in the lower chamber was counted.

### Immunoblotting

Immunoblots were performed as described in our previous study (Liu et al., 2015). Briefly, RIPA buffer was used to lyse cells and obtain proteins, which were separated by SDS-PAGE and transferred to PVDF membranes. After the proteins contained in PVDF membranes were blocked with TBS containing 5% skimmed milk, they were probed with the respective primary antibodies and HRP-conjugated secondary antibody for 2 h at room temperature, respectively. The blots were finally shown by Amersham Biosciences chemiluminescence.

### Immunofluorescence

After being fixed in 4% paraformaldehyde and washed by PBS, cells were incubated with indicated antibodies at 4°C overnight. The cells were washed by PBS twice and then probed with FITC-conjugated secondary antibodies at room temperature for 1 h. The slides were presented by a Leica fluorescence microscope.

### TOP/FOP Flash assay

In this study, we used the Upstate Biotechnology system to perform TOP/FOP Flash assay. In brief, after Fop-flash plasmid plus pRL-CMV plasmid (to normalize the transfection efficiency) or Top-flash plasmid plus pRL-CMV plasmid were transfected into the indicated cells for 48 h, a Dual Luciferase Kit (Promega) was used to measure the luciferase activity in cell extracts.

### Data analysis and statistics

Two-tailed Student's *t*-tests were used for single comparison and the log-rank test was used for multiple comparisons. Correlations in the gene expression levels were tested by the Spearman rank correlation. The Kaplan–Meier method was used to assess patients’ survival curve. Statistically significant results were shown as *P<0.05, **P<0.01 and ***P<0.001.

## Supplementary Material

Supplementary information
