# Correction: DJ-1 promotes epithelial-to-mesenchymal transition via enhancing FGF9 expression in colorectal cancer

**DOI:** 10.1242/bio.059782

**Published:** 2023-01-12

**Authors:** Longhao Li, Chundong Zhang, Yi Li, Ying Zhang, Yunlong Lei

There were errors published in *Biol. Open* (2020) **9**, bio051680. doi:10.1242/bio.051680.

**Fig. 2 (corrected).**
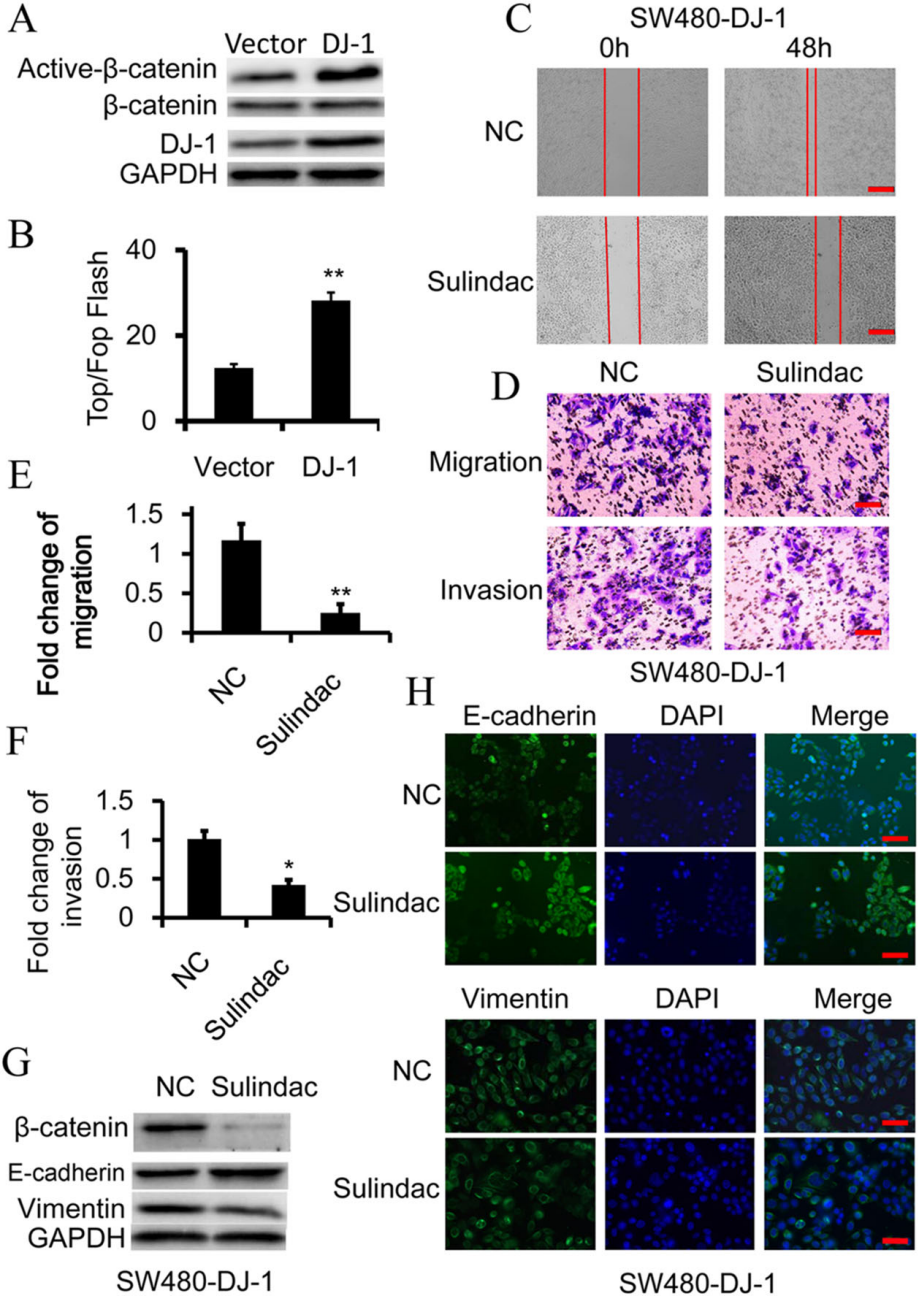
**Wnt signaling is essential for DJ-1-induced EMT.** (A) Western blot analysis of β-catenin and activated-β-catenin (non-phosphorylated β-catenin) expression in SW480 cells, in which DJ-1 was overexpressed. (B) TOP-Flash/FOP-Flash assay depicting Wnt signaling activity in SW480 and DJ-1-overexpressed SW480 (SW480-DJ-1) cells. (C-H) SW480-DJ-1 cells were treated with 100 μM Wnt inhibitor Sulindac for 36 h. (C) Wound-healing migration assay of indicated time. Scale bars: 250 μm. (D-F) Quantitative analysis of cell migration and Matrigel invasion assays. Migration was analyzed at 24 h, invasion at 48 h. All data were from at least three independent experiments and shown as mean±s.d. Scale bars: 50 μm. (G) Expression of β-catenin, E-cadherin, and Vimentin was examined by immunoblot. (H) Expression of E-cadherin and vimentin was examined by immunofluorescence. Scale bars: 50 μm. *P<0.05, **P<0.01.

In Fig. 2C, the image of Sulindac-treated SW480-DJ-1 cells (0h) was inadvertently misplaced with an image of SW480-Vector cells (0h). In addition, in Fig. 2H the image of Sulindac-treated SW480-DJ-1 cells (Vimentin and DAPI panels) was inadvertently misplaced with an image of siFGF9-treated SW480-DJ-1 cells.

**Fig. 2 (original).**
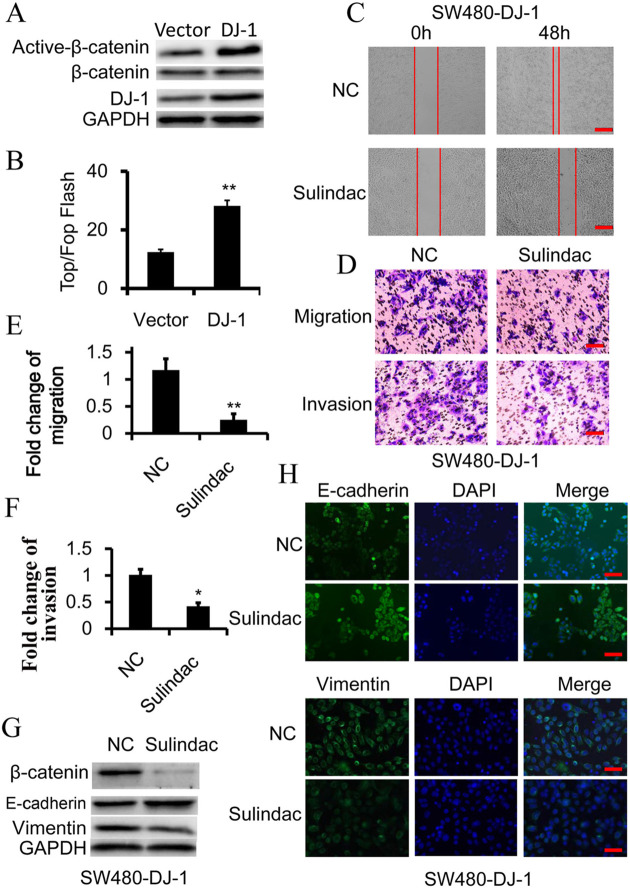
**Wnt signaling is essential for DJ-1-induced EMT.** (A) Western blot analysis of β-catenin and activated-β-catenin (non-phosphorylated β-catenin) expression in SW480 cells, in which DJ-1 was overexpressed. (B) TOP-Flash/FOP-Flash assay depicting Wnt signaling activity in SW480 and DJ-1-overexpressed SW480 (SW480-DJ-1) cells. (C-H) SW480-DJ-1 cells were treated with 100 μM Wnt inhibitor Sulindac for 36 h. (C) Wound-healing migration assay of indicated time. Scale bars: 250 μm. (D-F) Quantitative analysis of cell migration and Matrigel invasion assays. Migration was analyzed at 24 h, invasion at 48 h. All data were from at least three independent experiments and shown as mean±s.d. Scale bars: 50 μm. (G) Expression of β-catenin, E-cadherin, and Vimentin was examined by immunoblot. (H) Expression of E-cadherin and vimentin was examined by immunofluorescence. Scale bars: 50 μm. *P<0.05, **P<0.01.

The corrected and original figures are shown below. Both the online full-text and PDF versions of the article have been updated. The authors apologise to readers for this error, which does not impact the results or conclusions of this paper.

