# Supplementary material for: DJ-1 promotes epithelial-to-mesenchymal transition via enhancing FGF9 expression in colorectal cancer
Source: Biol Open. 2020 May 21;9(5):bio051680. doi: 10.1242/bio.051680 (PMC7325429; doi:10.1242/bio.051680)
Supplement: Supplementary information [file biolopen-9-051680-s1.pdf]

Table S1. Primers for qRT-PCR

Primers sequences for selected genes	
Gene	Sequences
DJ-1	Forward 5'-GGAGACGGTCATCCCTGTAG-3'
	Reverse 5'-TTCACAGCAGCAGACTCAGA-3'
E-cadherin	Forward 5'-TGAAGGTGACAGAGCCTCTGGAT-3'
	Reverse 5'-TGGGTGAATTCGGGCTTGTT-3'
Vimentin	Forward 5'-CCAAACTTTTCCTCCCTGAACC-3'
	Reverse 5'-GTGATGCTGAGAAGTTTCGTTGA-3'
FGF9	Forward 5'-GTGGACTCTACCTCGGGATG-3'
	Reverse 5'-GTGTGAATTTCTGGTGCCGT-3'

Biology Open • Supplementary information

Supplementary Figure1

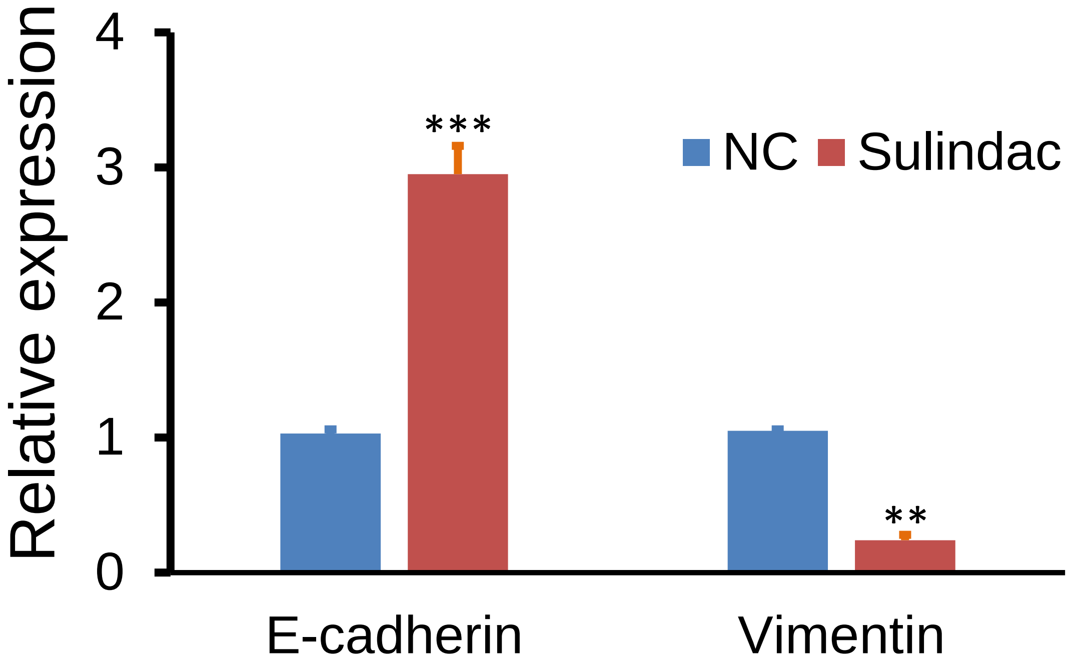

Figure S1 Wnt signaling is essential for DJ-1-induced transcription of EMT markers. SW480-DJ-1 cells were treated with 100 μM Wnt inhibitor Sulindac for 36 h. Expression of E-cadherin and Vimentin was examined by qRT-PCR. y axis means relative expression to control group. **, P < 0.01, ***, P < 0.001.

Supplementary Figure 2

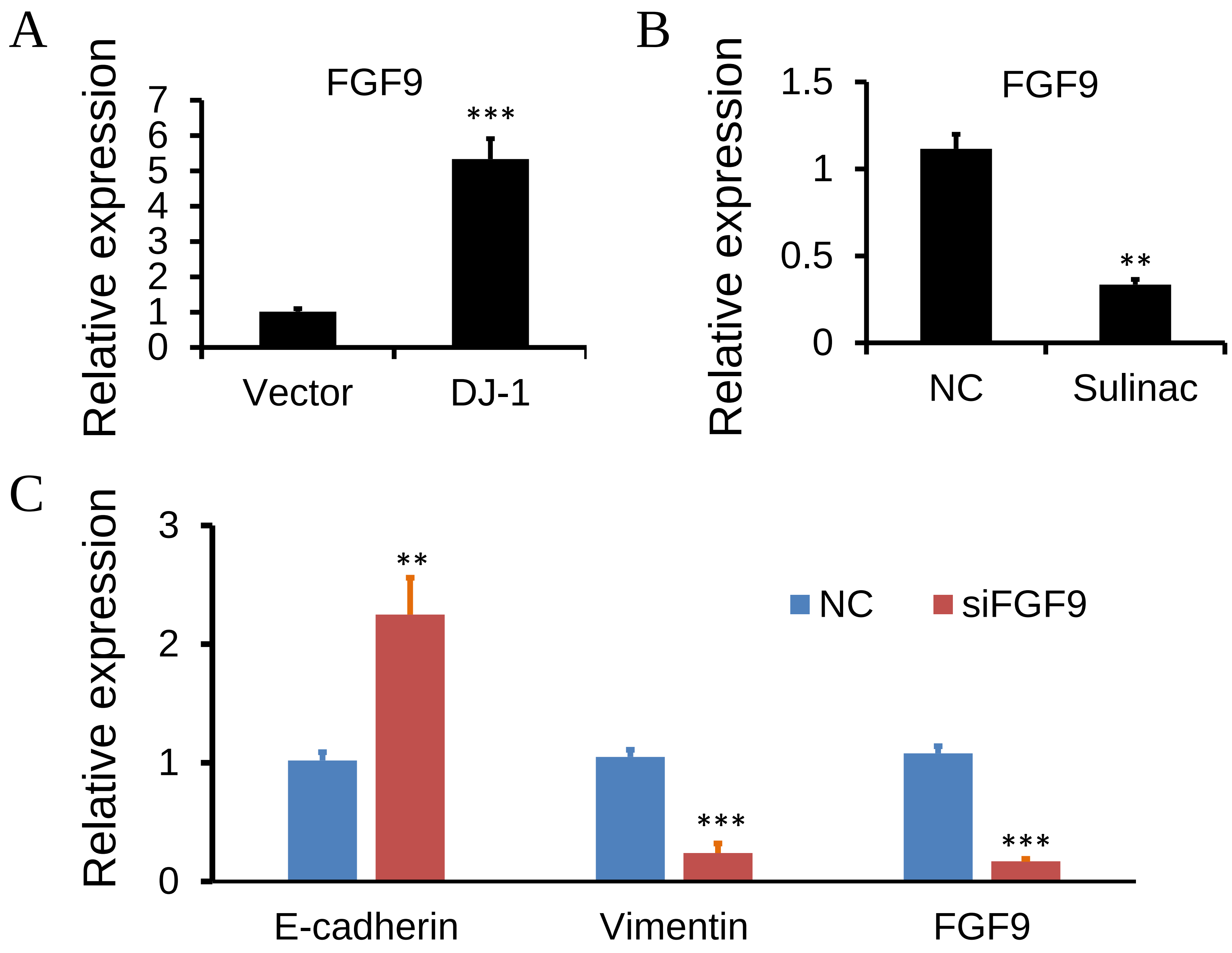

Figure S2 FGF9 is required for DJ-1-induced and Wnt signaling-mediated EMT. **A.** qRT-PCR analysis of FGF9 expression in SW480 cells stable transfected with DJ-1 cDNA or vector. **B.** qRT-PCR analysis of FGF9 in SW480-DJ-1 cells treated with or without 100 μM Sulindac for 36 h. **C.** SW480-DJ-1 cells were transfected with specific FGF9 siRNA. Expression of FGF9, E-cadherin and Vimentin was examined by qRT-PCR. y axis means relative expression to control group. **, $P < 0.01$, ***, $P < 0.001$.

Biology Open • Supplementary information

Supplementary Figure 3

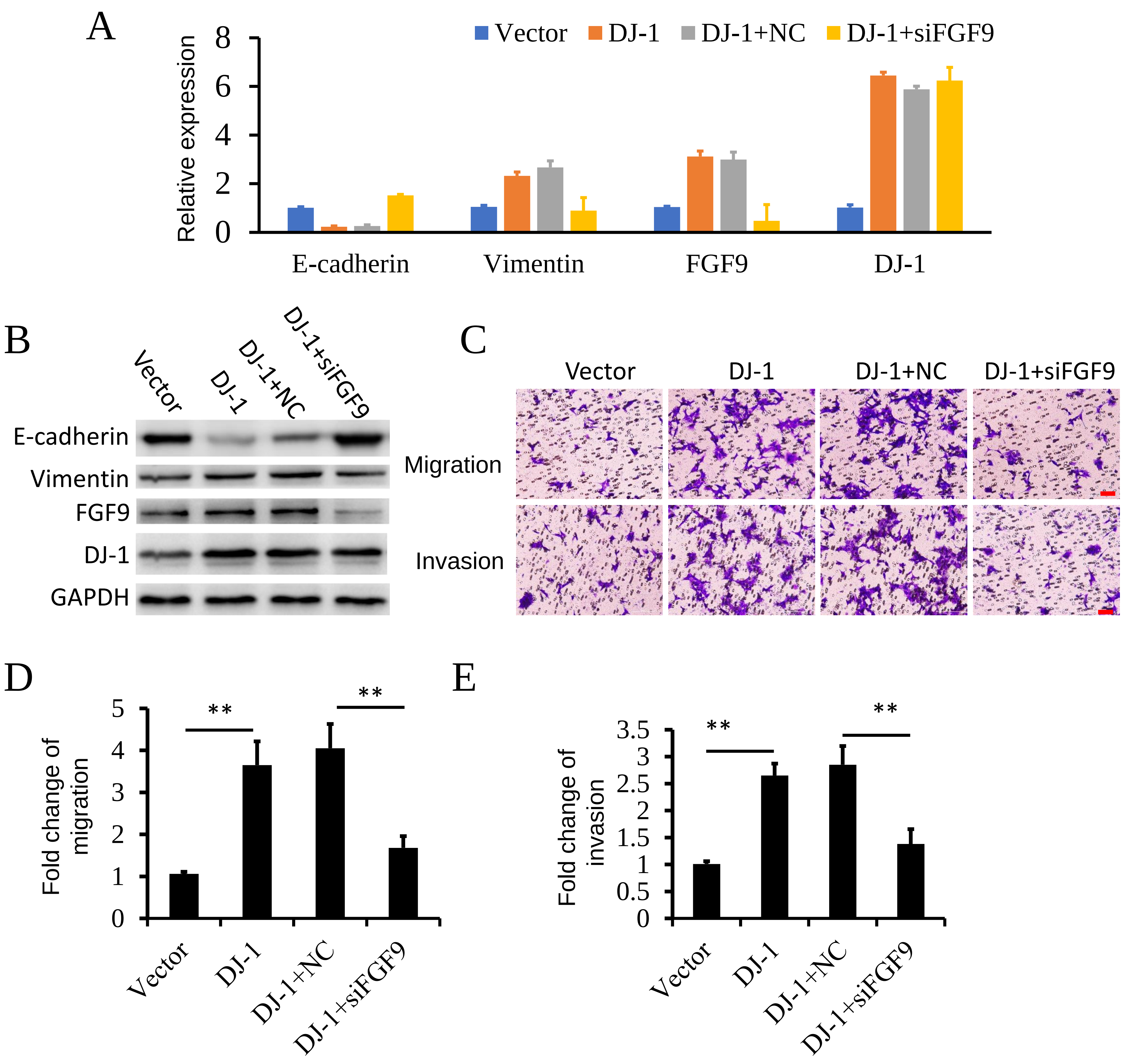

Figure S3 DJ-1 could promote EMT by upregulating FGF9 in RKO cells. RKO cells were transfected with DJ-1 or in combination for transfecting with siNC or siFGF9. **A.** Expression of DJ-1, FGF9, E-cadherin and Vimentin was examined by qRT-PCR. y axis means relative expression to control group. **B.** Expression of DJ-1, FGF9, E-cadherin, and Vimentin was examined by immunoblot. **C-E.** Quantitative analysis of cell migration and Matrigel invasion assays. Migration was analyzed at 24 h, invasion at 48 h. All data were from at least three independent experiments and shown as mean ± S.D. Scale bar, 50 μm.

Supplementary Figure 4

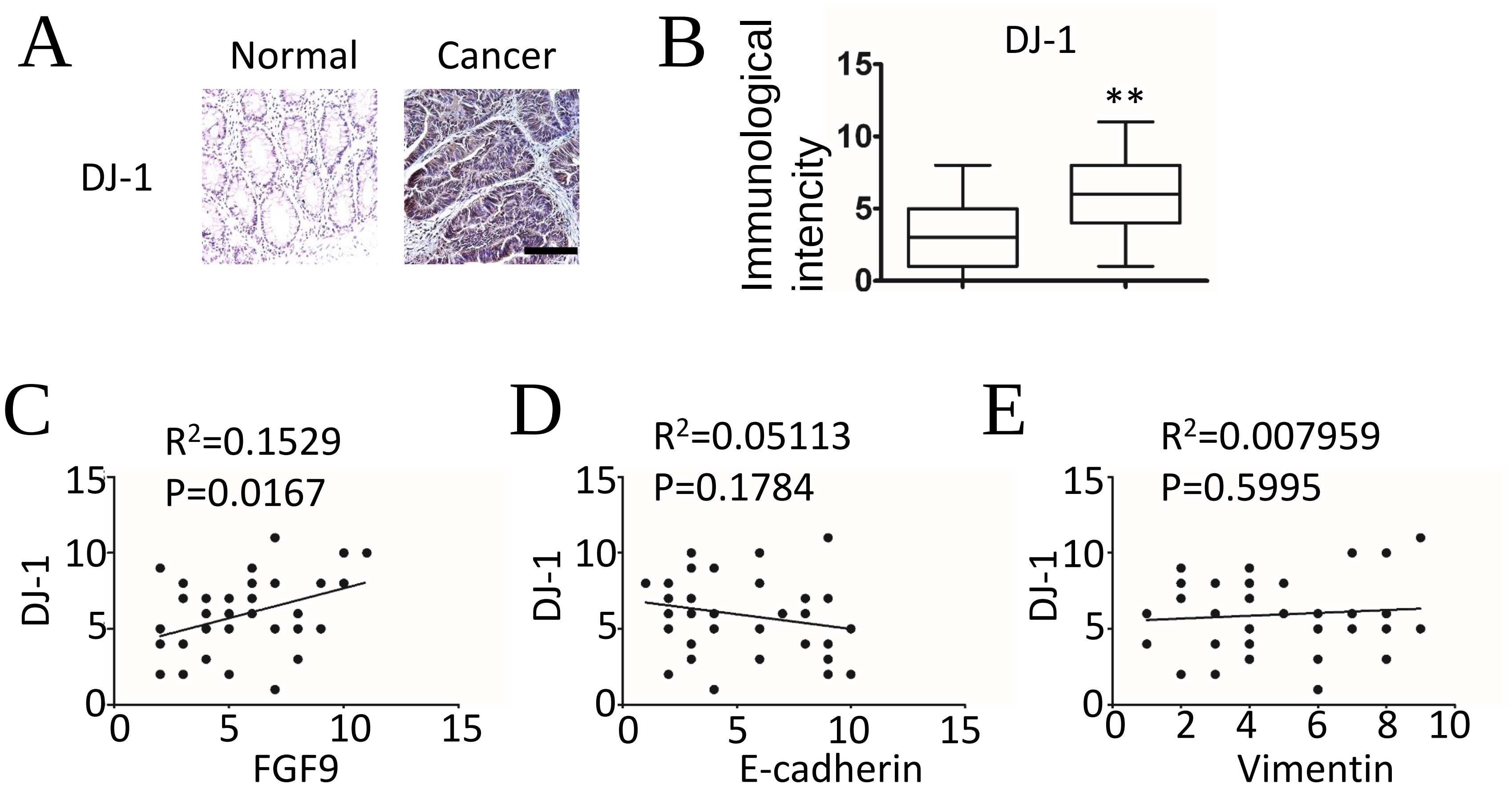

Figure S4 FGF9 is closely associated with DJ-1 expression in CRC patients. A. Immunohistochemical staining of DJ-1 in paraffin-embedded human CRC tissues. Scale bar, 25 µm. **B.** Immunohistochemical scores for DJ-1 in normal colorectal mucosa and CRC tissues (n=37). **C.** The correlation test of immunostaining intensity between DJ-1 and FGF9. **E.** The correlation test of immunostaining intensity between DJ-1 and E-cadherin. **F.** The correlation test of immunostaining intensity between DJ-1 and Vimentin. **, P < 0.01.

Biology Open • Supplementary information
